# Aluminium in Allergies and Allergen immunotherapy

**DOI:** 10.1186/s40413-015-0060-5

**Published:** 2015-02-28

**Authors:** Erika Jensen-Jarolim

**Affiliations:** Comparative Medicine, Messerli Research Institute, The University of Vet. Medicine Vienna, the Medical University Vienna, and the University Vienna, Währinger G. 18-20, 1090 Vienna, Austria; Institute of Pathophysiology and Allergy Research, Center of Pathophysiology, Infectiology and Immunology, Medical University Vienna, Vienna, Austria

**Keywords:** Allergen, Aluminium, Adjuvant, Allergen immunotherapy, Th2

## Abstract

Aluminium is a hot topic in the current debate. Exposure occurs due to environmental, dietary and intentional exposure to aluminium, such as in vaccines where it was introduced in 1926. In spite of the fact that it is a typical Th2 adjuvant, aluminium redirects the immune response in systemic allergen immunotherapy (SIT) upon prolonged immunization. SIT in the US, and SLIT in general, are at present non-adjuvanted therapies, but in Europe aluminium is used as adjuvant in most SIT preparations. It enhances the safety of SIT by local deposition of the allergen. Undesired properties of aluminium adjuvants comprise acute and chronic inflammation at the injection site, its Th2 immune stimulatory capacity, its accumulation besides biodistribution in the body. The adjuvant and safety profile of aluminium adjuvants in allergy vaccines are discussed, as well as the need for putting modern delivery systems and adjuvants on the fast track.

## Introduction

### When and why aluminium adjuvants were introduced into medicine

Glenny et al. reported the first time that aluminium, in a potassium salt form, could be of use in vaccine preparations in 1926, when it was induced to enhance the immunogenicity of toxoid preparations [1]. Later, several studies compared different aluminium compounds, especially focusing on aluminium hydroxide versus aluminium phosphate as adjuvants (Al_ADJ_). Since then the most important vaccines were formulated and tested with aluminium hydroxide, such as diphtheria toxin, [2], or diphtheria with tetanus toxoid [3]. Today most important vaccines approved by the US Food and Drug Administration are formulated with aluminium hydroxide [4] (Table 1), and at least for primary immunizations Al_ADJ_ has a superior activity, although may be less important for booster immunizations. Also in veterinary medicine aluminium hydroxide is in broad use (rev. by [5] (Table 2). Further, the depot effect of Al_ADJ_ was recognized, improved by formulation with monostearate and exploited in antibiotics applications against syphilis [6], gonorrhea [7] or other infections [8]. Last not least, aluminium hydroxide was introduced for the healing and prevention of gastric ulcers [9,10], bowel fistulas [11], and its superior proton buffer capacity as compared to other compounds was recognized experimentally in a dog model [12].

**Table 1:** Vaccines licenced in the US containing aluminum in adjuvants [4]

**Vaccines with aluminium adjuvant**	**Vaccines without aluminium adjuvant**
DTP (diphtheria-tetanus-pertussis vaccine)	Inactivated Polio Virus
DTaP (diphtheria-tetanus-acellular pertussis vaccine)	Measles vaccine
Hib (Haemophilus influenzae type b) conjugate vaccines (not all)	Mumps vaccine
Pneumococcal conjugate vaccine	Rubella vaccine (MMR),
Hepatitis B vaccines	Varicella vaccine
All combination DTaP, Tdap, Hib	Influenza vaccines
Hepatitis B vaccines	
Hepatitis A vaccines	
Human Papillomavirus vaccine	
Anthrax vaccine	
Rabies vaccine

**Table 2:** **Vaccines licensed for the use in animals containing aluminum (adapted from** [5]**)**

**Viral vaccines**	**Bacterial vaccines**
Avian infectious bronchitis virus	Bacteriodes nosodus
Canine hepatitis virus	Bordetella bronchispetica
Foot-and-mouth Disease	Clostridia (diverse)
Newcastle Disease Virus	Leptospira interrogans
	Pasteurella multocida

## Review

### Mechanism of aluminium adjuvants in systemic allergen immunotherapy

Type I allergy is characterized by specific IgE, a typical Th2 skewing associated with IL-4, IL-13 cytokines, sometimes accompanied by eosinophilic inflammation. It is understood today that systemic allergen immunotherapy (SIT) leads to a counterbalance of Th2 type immunity by enhancement of Th1 responses and/or induction of immune tolerance by T- and B-regulatory cells, accompanied by IL-10, IFNγ and TGFβ production [13,14]. Systemic allergen immunotherapy (SIT) is the only causative treatment available today. It is a long-term treatment over years and tackles the problem via both, the subcutaneous (SCIT) or sublingual (SLIT) routes [15]. It is also known that often in the onset of SIT IgE levels are even enhanced when Al_ADJ_ are used, whereas prolonged immunization lead to “modified Th2 immunity” with protective character. Although, there is still no consensus about a downregulation of specific IgE, it is accepted since long that the induction of IgG, especially the non-inflammatory IgG4, is a hallmark of allergen immunotherapy [16]. No convincing data are available today that indicate that any of the changes in antibody subclass, of the investigated cytokines or factors do correlate with the clinical outcome and can be regarded as a biomarker.

In fact, when Noon and Freeman experimented in 1911 with the inoculation of allergen extracts as prophylactic vaccines [17,18], they did not use aluminium compounds. Al_ADJ_ was first introduced into allergy vaccines in 1937 [19]. Since, several authors investigated different aluminium forms, such as aluminium hydroxide (Al(OH)_3_), aluminium phosphate (AlPO_4_), or aluminium monostearate [20], with focus on precipitation capacity and depot effects of the adjuvants [21]. Today, Al_ADJ_ are used as adjuvant in European allergy vaccines, whereas in the US allergy vaccines are adjuvant free and soluble [22].

The boost of allergy by immunizations with Al_ADJ_ was well-known even since the time before the discovery of the IgE immunoglobulin class, and was studied in animal and human models [23-26]. It was also recognized that vaccination with an independent antigen with aluminium could pre-determine the type of immunity to a consecutive antigen. For instance, rats that were immunized with Pertussis toxin and Al(OH)_3_ showed an increased “reaginic response” (IgE) to the allergen ovalbumin [27]. This “non-specific enhancement of allergy” by a vaccine was later confirmed in rats but the authors showed that the effect was independent on aluminium adjuvant [28].

When (still in 1972) young healthy men were experimentally immunized with allergoids of rye grass pollen group 1 allergens in combination with Al_ADJ_, they developed type I skin hypersensitivity, specific histamine releasability, and passively transferable IgE antibodies [29]. The study subjects, however, did not develop clinical symptoms, possibly due to the fact that also “agglutinating antibodies” (IgG) were formed, which according to todays’ point of view might act as blocking antibodies. After this human model it was shown in a dog model, that sensitization to 2,4-dinitrobenzene conjugated to ovalbumin led to IgE formation, which was associated with bronchial constriction upon allergen challenge when the IgE levels were only high enough [30]. It was reported in the same year, that the immune response in other mammalians may differ: when cows were treated by a vaccine to foot-and-mouth disease virus formulated in Al(OH)_3_ they produced reaginic and hemagglutinating antibodies, but there was no correlation of each parameter to the size of the intradermal test reaction [31].

Itaya and collegues confirmed in mice that aluminium compounds were excellent adjuvants for IgE induction, whereas other adjuvants including LPS were not. Interestingly, when the adjuvants including Al_ADJ_ were given before sensitization, they suppressed a consecutive allergic response [32].

Further, it was reported that in rabbits i.m. injections with both aluminium hydroxide and aluminium phosphate led to increased Al levels in the blood already after 1 hour, and that after 28 days 3x more of the Al(OH)_3_ remained absorbed in the body, in the following tissues: kidney > spleen > liver > heart > lymph node > brain [33].

Today, most subcutaneous allergen immunotherapy is performed with Al(OH)_3_ as adjuvant, less by calcium phosphate, but none of the SLIT preparations contains any adjuvants [22]. Other formulations, like probiotics, mycobacteria (attenuated or ghosts), virosomes, TLR ligands, cochleates, proticles, etc. are still in the experimental stage [34,35].

### Effects and side effects of aluminium in allergy vaccination

When using Al_ADJ_ for formulating allergens for SIT, prolonged immunization leads to the induction of allergen-specific IgG which finally dominates the IgE response [36]. The need for prolonged immunizations in SIT may be due to the difficulty to modify an already established Th2 response, whereas it is easier to induce protective immunity by prophylactic vaccine. The induced IgG dampen the allergic response via inhibiting the IgE-allergen interaction and associated facilitated allergen presentation [37], and they compete with IgE by quantity rather than quality: the affinity of IgG to important allergens like Bet v 1 and Phl p 5, is significantly lower than that of IgE [38]. The following major features are attributed to Al_ADJ_: i) in vaccine formulations at neutral pH aluminium compounds are positively charged and absorb negatively charged proteins by electrostatic mechanism of which the strength will depend on the avidity of interaction [39]; ii) Al_ADJ_ via adsorption and entrapment formulates the allergen also into nano- or microparticles which are preferentially taken up by phagocytes through innate mechanisms involving mast cells and macrophages, and may involve the inflammasome [40-42]. This leads to an immediate release of inflammatory Th1 and Th2 cytokines, with endogenous IL-18 facilitating IL-4 production [43]. The immunomodulatory properties that include the innate and adaptive branch of the immune system are reviewed in great detail in [44]; iii) Al_ADJ_ increase phagocytosis of the allergen by DCs, which interestingly in the absence of DC activation leads to antigen presentation after 6 h [45]; iv) Al_ADJ_ precipitate the allergen, forming a depot [1], from where the allergen is released slowly [39]. The local deposition prevents immediate release of allergens in the hypersensitive patient and contributes to safety of Al_ADJ_.

Surprisingly, recently Al(OH)_3_ in comparison to the Th1 adjuvant Montanide induced almost the same humoral immune response to Adeno-associated virus-like particles, except that the formation of IgG2a and IgG2b were more pronounced by Montanide [46]. In earlier studies Al_ADJ_ was compared head-to-head to other adjuvants and showed superior effects when used in a ragweed pollen [47] or birch pollen vaccine [48]. Given the paradox fact that Th2-biasing Al_ADJ_ in SIT are used to cure a Th2 type disease, from the immunological point of view it is astonishing that these adjuvants can achieve the observed high efficacy [39]. Still, the outcome of SIT could be improved by avoiding boosting of IgE and by redirecting the immune system more effectively, and possibly this altogether could shorten its duration. It has therefore been suggested that allergen immunotherapy could be improved by addition of immunopotentiating substances redirecting the immune reactivity to Th1, or being immunomodulatory by their particulate nature [49], pronounced TLR binding capacity [50], or through their muco-adhesive properties [22]. Important to note that in preclinical studies most often prophylactic models are used which do not resemble the setting in an already sensitized patient with flowering Th2 immune response. Therefore, for proof of concept studies rather therapeutic models should be favored. In fact, in a therapeutic mouse model an oral vaccine based on grass pollen allergens entrapped in microparticles was able to modify an already established allergic response when the vaccine was targeted to mucosal M-cells. This approach was independent on usage of Al_ADJ_ [51]. Alternative adjuvants may also be needed when in addition to high antibody levels also induction of cytotoxic T-cell responses and a higher degree of antigen presentation are desired [52], such as in cancer vaccines.

Only minor side effects have been reported so far for allergy vaccines [53] in context with Al_ADJ_, such as local pruritic nodules which may be acute and transient in 33-70% of injections [54], and which sometimes persist as granules [55,56]. Generally it is believed that the local inflammation due to a vaccine shot is important for induction of an efficient immune response. More recently, the induction of contact dermatitis to aluminium itself upon vaccination could be proven by skin testing in 5/78 children and 3/127 adults [57].

### Aluminium exposure, dosage and undesired effects

Aluminium is an abundant compound in our environment in the “aluminium age” [58]. Many novel materials are imprinted with nanoparticulate aluminium to promote their comfort of use, such as textiles or toothpaste. Still, the physical and chemical forms of aluminium determine its bioavailability and hence toxicity. In the insoluble form aluminium compounds may form particles, which lead to inflammation upon ingestion or respiration.

The intakes in drinking water vary largely [59] but may in some areas exceed 15 times the World Health Organization recommendations for tolerable weekly intake (TWI). The solubility depends on the pH and may at acidic pH even reach 90 mg/L. It may be airborne at levels between 0.0005 μg/m^3^ (arctic levels) to 1 μg/m^3^ (industrial area) [60]. Also food and feed-intake and -additives contribute to aluminium consumption. Altogether, the European Food Safety Agency determined the TWI to 1 mg/kg body weight from all sources of aluminium [61]. Breast milk contains 0.04 mg/L aluminium. Therefore, until the age of 6 months breast-fed infants have consumed 7,2 mg aluminium totally, formula-fed 38 mg and soy-fed up to 112 mg, because soy is a plant that accumulates aluminium [62].

A hot topic in the current debate is that aluminium is introduced into the body by vaccines. In the US 0.85, and in cases of documented efficacy up to 1.25 mg per single vaccine shot are recommended [63], in Europe up to 1.25 mg [4]. For instance, vaccination patient information platforms put this amount in relation to 4.4 mg derived from vaccines during the same time slot in a baby’s life [64]. In SCIT with up to 54 injections during the whole course the accumulating dose may vary between 45 and 67,5 mg of aluminium [53].

Upon injection, in the tissues all possible forms of aluminium, including ions, soluble aluminium, particulate forms, alone or bound to antigen or tissue compounds can be found [44]. The injected Al_ADJ_ releases the biologically active form Al^3+^ and aluminate (Al(OH)_4_^−^) ions, which may react with water and finally lead to Al superoxide production [65]. However, most of the injected aluminium will be phagocytized and thereby activate cells that recruit even more inflammatory cells. Due to a high binding affinity with iron, aluminium intracellularly can deplete the mitochondria from Fe and lead to the production of reactive oxygen species (ROS). Aluminium can induce DNA damage through ROS and has an apoptotic effect. This has been shown for peripheral lymphocytes, which are susceptible especially in the G0/G1 phase of the cell cycle [66]. However, the amount of soluble Al locally after a vaccine injection may be insufficient to induce cell death [44]. It might be considered that aluminium-containing vaccines expose children in an age when both, immune function and brain development are sensitive [67].

**Figure 1:**
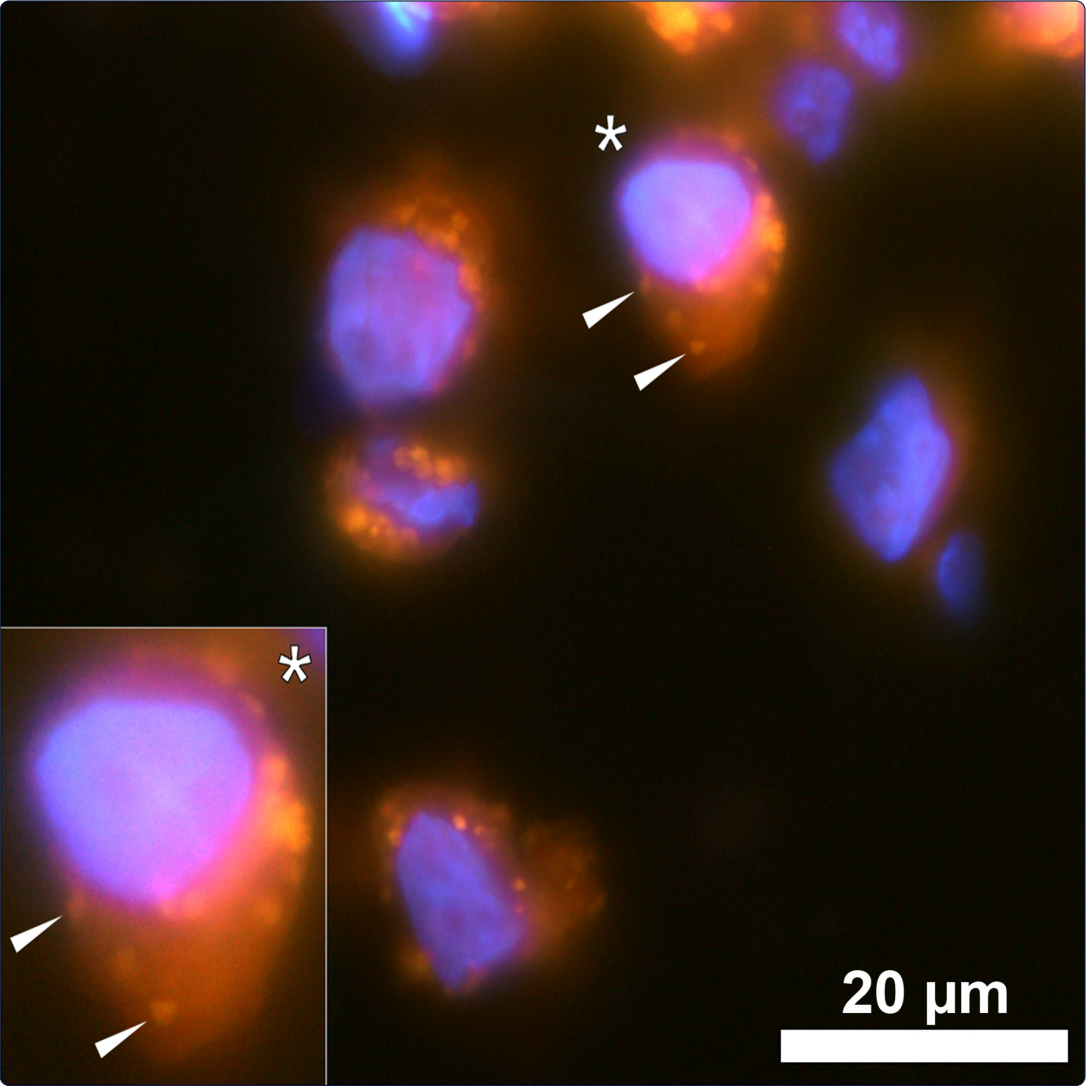
**Representative lumogallion staining of cryosectioned (6 μm section) THP-1 cells co-cultured with 50 μg/mL AlO(OH) Brenntag adjuvant for 24 h.** Cell sections were incubated for 24 h in 100 μM lumogallion, 50 mM PIPES, pH 7.4. Slides were mounted with ProLong® Gold Antifade Reagent with DAPI. Lumogallion (orange) and DAPI-staining (blue) is depicted. The insert shows a close-up of an individual cell and white arrows highlight distinguishable adjuvant particles. Magnification X 1000. Kindly provided by Dr. Matthew Mold and Dr. Chris Exley, Birchall Centre, Keele, UK.

The bidirectional exchange of cytokines and factors between brain and immune system has been recognized [68], and also phagocytosis of aluminium particles and trafficking of these cells from muscle into the brain has been reported [69]. Hence it is not surprising that aluminium intake has been discussed in context with neurologic disorders since a long time, especially Alzheimer. Ferritin represents a major storage of iron in mammalians and it has been recognized recently that aluminium may replace iron within this complex [70]. By mass spectrometry the authors found that the aluminium content in ferritin was higher in Alzheimer patients and depended on the disease stage. The enhanced intake via drinking in some geographical areas has been associated with the development of Alzheimer [59]. However, taken the available data together, the evidence seems to this end not entirely convincing [65], which to a part might be caused by methodological limitations. A specific aluminium-staining methodology was lacking so far and has only recently been developed using a fluorescent molecular probe for aluminium, lumogallion [71] (Figure 1).

One should remember that also orally taken antiacid drugs or sucralfate contain significant amounts of aluminium compounds. We could show that oral intakes of these aluminium compounds support Th2 sensitization to food proteins in mouse models of food allergy [72,73].

More recently, the so-called macrophagic myofasciitis has been attributed to the persistence of aluminium salts at injections sites in muscle [53], also chronic fatigue syndrome [74] and autoimmune diseases have been associated with aluminium intake, being termed ASIA – Autoimmune/inflammatory syndrome induced by adjuvants [75]. The groups with elevated risk include, besides patients with a previous history of autoimmunity, patients with allergy according to a recent paper [76].

In a comprehensive review on the present topic it was therefore suggested to refine the TWI aluminium dose, to reduce aluminium in parenteral solutions for infants (where it led to defects in bone mineralization), to harmonize occupational doses and to reconsider its use in vaccines [65].

## Conclusion

Aluminium has been used since 1926 in human and veterinary medicine and since 1937 in allergy and can generally be regarded as safe in terms of acute local or systemic side effects. The reports on chronic toxicity of aluminium, however, including ASIA are accumulating and are discussed seriously by national authorities, for instance as France [77], Austria [78], or the US [63]. Therefore, it is proposed here that novel and promising immunomodulators and allergen delivery systems that are in the pipeline should be put on the fast track.
